# Single-Cell Genomics Reveals a Diverse Metabolic Potential of Uncultivated *Desulfatiglans*-Related Deltaproteobacteria Widely Distributed in Marine Sediment

**DOI:** 10.3389/fmicb.2018.02038

**Published:** 2018-09-03

**Authors:** Lara M. Jochum, Lars Schreiber, Ian P. G. Marshall, Bo B. Jørgensen, Andreas Schramm, Kasper U. Kjeldsen

**Affiliations:** Center for Geomicrobiology, Section for Microbiology, Department of Bioscience, Aarhus University, Aarhus, Denmark

**Keywords:** single cell genomics, *Desulfarculaceae*, acetogenesis, dissimilatory sulfate reduction, aromatic compounds degradation, dehalogenation, *rdhA*, marine sediments

## Abstract

*Desulfatiglans*-related organisms comprise one of the most abundant deltaproteobacterial lineages in marine sediments where they occur throughout the sediment column in a gradient of increasing sulfate and organic carbon limitation with depth. Characterized *Desulfatiglans* isolates are dissimilatory sulfate reducers able to grow by degrading aromatic hydrocarbons. The ecophysiology of environmental *Desulfatiglans*-populations is poorly understood, however, possibly utilization of aromatic compounds may explain their predominance in marine subsurface sediments. We sequenced and analyzed seven *Desulfatiglans*-related single-cell genomes (SAGs) from Aarhus Bay sediments to characterize their metabolic potential with regard to aromatic compound degradation and energy metabolism. The average genome assembly size was 1.3 Mbp and completeness estimates ranged between 20 and 50%. Five of the SAGs (group 1) originated from the sulfate-rich surface part of the sediment while two (group 2) originated from sulfate-depleted subsurface sediment. Based on 16S rRNA gene amplicon sequencing group 2 SAGs represent the more frequent types of *Desulfatiglans*-populations in Aarhus Bay sediments. Genes indicative of aromatic compound degradation could be identified in both groups, but the two groups were metabolically distinct with regard to energy conservation. Group 1 SAGs carry a full set of genes for dissimilatory sulfate reduction, whereas the group 2 SAGs lacked any genetic evidence for sulfate reduction. The latter may be due to incompleteness of the SAGs, but as alternative energy metabolisms group 2 SAGs carry the genetic potential for growth by acetogenesis and fermentation. Group 1 SAGs encoded reductive dehalogenase genes, allowing them to access organohalides and possibly conserve energy by their reduction. Both groups possess sulfatases unlike their cultured relatives allowing them to utilize sulfate esters as source of organic carbon and sulfate. In conclusion, the uncultivated marine *Desulfatiglans* populations are metabolically diverse, likely reflecting different strategies for coping with energy and sulfate limitation in the subsurface seabed.

## Introduction

Subseafloor sediments harbor more than half of the oceans’ microorganisms (Kallmeyer et al., 2012; Parkes et al., 2014). Subseafloor microbial communities are buried in the sediment over time due to sedimentation. During burial they experience a successive change in geochemical conditions and increasing organic carbon limitation with sediment depth (Middelburg, 1989; Canfield and Thamdrup, 2009; Langerhuus et al., 2012; Lomstein et al., 2012). The decreasing availability of organic carbon is arguably a severe restriction on the activity, size, and composition of microbial communities in subsurface sediments (Lever et al., 2015; Petro et al., 2017). The most labile compounds are consumed near the seafloor (Middelburg, 1989; Boudreau and Ruddick, 1991) and consequently, microorganisms in deeper layers have to rely on more recalcitrant organic matter for sustenance and growth (Middelburg, 1989; Biddle et al., 2006). Therefore, the capacity of a given microbial population to use organic material of low reactivity or in other ways cope with the energy limitation of subsurface sediments expectedly control if the population survives in this environment. Regardless of geography, certain microbial taxonomic groups tend to dominate subsurface sediments (Orcutt et al., 2011; Parkes et al., 2014) suggesting that they must possess adaptations to subsurface life. Starnawski et al. (2017) concluded that such adaptations are present in a population before being buried in subsurface sediments, as the number of generations that microorganisms undergo during burial into deeper sediment layers likely does not allow for *in situ* adaptive evolution. Adaptations that allow microorganisms to survive under increasing energy limitation are, however, poorly understood and remain a challenge to investigate.

Sulfate-reducing microorganisms (SRMs) are key players in the marine carbon and sulfur cycles (Jørgensen, 1982). Profiling of phylogenetic and functional marker genes for SRM showed that they populate both the upper sulfate-rich part and the deeper sulfate-depleted parts of marine sediments (Leloup et al., 2007, 2009; Jochum et al., 2017). In Aarhus Bay subsurface sediments a group of uncultured Deltaproteobacteria phylogenetically related to members of the sulfate-reducing genus *Desulfatiglans* is particularly abundant (Starnawski et al., 2017). Similarly, *Desulfatiglans*-related populations constitute one of the most abundant taxonomic groups of known SRM in arctic, temperate, and tropical sediments (Robador et al., 2016) suggesting a significance of this group for the global marine sulfur cycle. The genus *Desulfatiglans* is comprised of a few cultured isolates and stable enrichment cultures and a large number of environmental sequences. The cultured isolates stem from marine and freshwater surface sediments and were enriched and isolated either from sediments contaminated with aromatic compounds (Selesi et al., 2010; Suzuki et al., 2014) or selectively enriched on aromatic compounds from pristine sediments (Schnell et al., 1989; Galushko et al., 1999; Ahn et al., 2009; Musat et al., 2009). All known enrichments or isolates of the genus *Desulfatiglans* are capable of growing by degrading monocyclic and/or polycyclic aromatic compounds (Schnell et al., 1989; Galushko et al., 1999; Ahn et al., 2009; Musat et al., 2009; Selesi et al., 2010; Suzuki et al., 2014). Aromatic compounds increase in relative abundance in the characterizable organic matter pool in subsurface marine sediments relative to surface sediments (Oni et al., 2015), which could explain the abundance of *Desulfatiglans*-related taxa in marine sediments. Anaerobic degradation of aromatics requires the presence of several specific enzymes (Fuchs et al., 2011) and should therefore not be available to the majority of subseafloor SRM, which are typically specialized in the oxidation of low-molecular weight organic compounds and H_2_ (Muyzer and Stams, 2008). Depending on the enzymatic pathway, the reduction of the aromatic ring can require investment of ATP (Fuchs et al., 2011), which may render sulfate-dependent aromatic compound degradation an unfavorable catabolic strategy in energy-depleted subsurface sediments. Correspondingly, *Desulfatiglans anilini* (*D. anilini*) possesses genes encoding the enzymes of an ATP-independent pathway (Kuntze et al., 2011), while enzymes of both ATP-dependent and ATP-independent aromatic compound degradation are encoded in the genome of the related sulfate-reducing strain NaphS2 (DiDonato et al., 2010). The metabolic potential and ecology of uncultivated *Desulfatiglans*-related bacteria inhabiting marine sediments have so far not been investigated and their capacity for aromatic compound degradation is not known.

The subseafloor *Desulfatiglans* populations offer a rare possibility to compare environmentally predominant microorganisms to cultured close relatives. We sequenced and analyzed the genomes of seven single cells affiliated with the genus *Desulfatiglans* obtained from different sediment depths of Aarhus Bay, Denmark and compared them to their closest cultured relatives with fully sequenced genomes, *D. anilini* and the sulfate-reducing strain NaphS2. The overall goal of the study was to identify adaptations to subseafloor life in environmental *Desulfatiglans* populations with regard to carbon and energy metabolism. Our specific objective was to identify whether sulfate-dependent aromatic compound degradation was present and widespread in the environmental *Desulfatiglans* populations along with genetic features that distinguish them from their cultured relatives and could convey advantages in the subseafloor environment. Our approach allowed us to identify differences and commonalities between the uncultured Aarhus Bay sediment *Desulfatiglans* populations and their cultured relatives, pointing to a metabolic diversity so far not characterized for members of this genus. We found evidence for aromatic compound degradation and sulfate reduction, yet they appear to be metabolically versatile as one group of exclusively environmental origin does not appear possess the genetic potential for dissimilatory sulfate reduction. *Desulfatiglans*-related organisms thus seem to maintain high abundances throughout the vertical sediment column by being able to use a wide range of organic carbon substrates and shift between different types of energy metabolism.

## Materials and Methods

### Sampling and Cell Extractions

Sediment was sampled from station M5 (Langerhuus et al., 2012) and station Mimosa (Lloyd et al., 2013) (**Supplementary Table S1**) in Aarhus Bay by Rumohr Lot and gravity coring. Sediment subsamples for cell extraction were taken from intact core material stored at 4°C for less than 24 h after coring. Cells were extracted from 25 cm and 175 cm below seafloor at station M5 and 10 cm below seafloor at station Mimosa (**Supplementary Table S1**). Cells for single-cell sorting were extracted from the sediment by density gradient centrifugation and prepared for single-cell sorting as previously described (Lloyd et al., 2013).

### Generation of Single Cell Amplified Genomes

Single-cell sorting, whole-genome amplification and 16S rRNA gene PCR screening of single cells were performed at the Single Cell Genomics Center at Bigelow Laboratory for Ocean Sciences, Maine, using their established protocols as previously described for the cells used in this study (Lloyd et al., 2013; Starnawski et al., 2017). The sorted cells were lysed by combining freeze-thaw cycles with cold KOH lysis treatment. The genomic DNA from the lysed cells was amplified by multiple displacement amplification at 30°C for 12–16 h (detailed Methods can be found in Lloyd et al., 2013). Barcoded sequence library preparation from single-cell amplified genomes (SAGs) was done by the Nextera XT DNA Library Preparation Kit (Illumina), with 1 ng of input DNA. The sequencing was performed on a MiSeq instrument with paired-end 2x 300 bp chemistry (Starnawski et al., 2017).

### SAG Assembly and Analysis

Sequence reads from the SAGs were processed with the trimmomatic software v0.32 (Bolger et al., 2014) for adapter trimming and quality filtering (flags used: Paired End [PE], ILLUMINACLIP:adapters.fa:2:40:15 for the adaptor trimming and SLIDINGWINDOW:4:20 for the quality filtering). SAGs were assembled using SPAdes v3.6.0 (Bankevich et al., 2012) with the –careful flag. Genome size was estimated by comparing the number of tRNAs of the two close cultured relatives of the SAGs with fully sequenced genomes (*D. anilini* and NaphS2) to the number of tRNAs found in the SAGs, as well as by counting the number of single copy genes shared by the two cultures that were also present in the SAGs using quickCOAT (Marshall et al., 2017a). Additionally, genome completeness and contamination were estimated using CheckM with the lineage_wf approach (Parks et al., 2015). By this approach, CheckM automatically finds the best taxonomic lineage to consider for genome completeness estimates via a set of common marker genes. Annotation was performed using the Integrated Microbial Genome (IMG) annotation service (Markowitz et al., 2014). Pathways and metabolic functions were identified based on the IMG annotation and supplemented by manual curating. The SAG sequences were compared to the annotated genome sequences of *D. anilini* (Suzuki et al., 2014) and deltaproteobacterium strain NaphS2 (DiDonato et al., 2010), as available on IMG (Taxon ID 2526164742 and 648276759, respectively). Average amino acid identity (AAI) between the SAGs was calculated using the enveomics online tool^1^ with a 50% identity cutoff (Rodriguez-R and Konstantinidis, 2016).

### Reductive Dehalogenase Subunit α (*rdhA*) Gene Phylogeny

Gene products representing RdhA domain homologs in the SAGs and the genome of the deltaproteobacterial strain NaphS2 were used as queries in blastp searches against the NCBI nr protein database and hits to *rdhA* gene products from known organohalide respiring organisms were retrieved as well from *Dethiosulfatarculus sandiegensis* for which organohalide respiration has so far not been experimentally validated. However, this isolate is the only other *Desulfarculaceae* member carrying *rdhA* gene homologs besides the SAGs and strain NaphS2. Protein sequences were aligned with the online version of T-coffee v8.93^2^ and default settings (Notredame et al., 2000) and based on 614 conserved alignment positions a phylogenetic tree was calculated using RAxML v8.2.9 (Stamatakis, 2014) with the PROTGAMMALGF model of evolution. Fe–S cluster binding motifs were identified with the online version of MetalPredator (Andreini et al., 2011; Valasatava et al., 2016).

### Distribution of *Desulfatiglans*-Affiliated Phylotypes in Aarhus Bay Sediments and SAG Phylogeny

We identified the 10 most abundant OTUs (operational taxonomic units, defined by a 97% sequence identity cutoff) classified as *Desulfatiglans* at the genus level in two existing 16S rRNA amplicon sequencing datasets (BioProject accession numbers PRJNA308429 and PRJNA377833) covering five different sediment sampling stations in Aarhus Bay (Marshall et al., 2017b; Starnawski et al., 2017). The sediment depth distribution of these OTUs was inferred from their relative abundance within the sequence libraries. The sequences were clustered into OTUs using the Vsearch algorithm (Rognes et al., 2016) as implemented in the mothur software (Schloss et al., 2009) and classified against the Silva SSU Ref NR99 v128 database (Quast et al., 2013) using the classify.seq command in mothur. The taxonomic affiliation of the identified OTUs was confirmed by phylogenetic analysis. The phylogenetic position of the 16S rRNA gene sequences of the SAGs within the family *Desulfarculaceae* was inferred by maximum likelihood analysis using RAxML v8.2.9 (Stamatakis, 2014) with the GTRGAMMA model of evolution. The sequences were aligned using the SILVA Incremental Aligner (SINA) v1.2.11 (Pruesse et al., 2012; Quast et al., 2013). Representative sequences (identified using the get.oturep command in mothur) of the 10 most abundant *Desulfatiglans*-affiliated OTUs from the Aarhus Bay sediment 16S rRNA gene amplicon sequence libraries were subsequently added to the maximum likelihood tree with the “Quick add marked” function of the ARB program package (Ludwig et al., 2004) along with short 16S rRNA gene sequences of SAG2 and 11 without changing the overall topology of the tree.

## Results and Discussion

### Single Cell Genome Assembly

The assembly size of the SAGs ranged from 826 kbp (SAG5) to 2.5 Mbp (SAG3) with 848 and 2,662 identified encoded genes, respectively (**Table 1**). The size of the genomes of *D. anilini* and strain NaphS2 is 4.6 and 6.5 Mbp, respectively. The difference between the genome size of the cultured isolates and the SAGs is at least in part due to incomplete recovery of SAGs. Completeness estimates based on the number of tRNAs ranged from 15% for SAG2 and SAG13 to 55% for SAG3 (**Table 1**). Similar levels of completeness were inferred by comparing the presence of single copy genes in the SAGs (**Table 1**). The degree of inferred completeness of prokaryotic SAGs typically varies substantially. For example, Rinke et al. (2013) reported completeness estimates ranging from 10 to 90% with an average of 40% in a study including 201 SAGs, while other studies report values between 35 and 85% (Kaster et al., 2014) and 32 and 70% (Lloyd et al., 2013). The incompleteness is likely caused by uneven genome amplification during multiple displacement amplification of single cell genomes (Clingenpeel et al., 2015). Calculating the total genome size of the SAGs from the CheckM genome completeness estimates (**Table 1**) give values between 4.3 and 6.2 Mbp, which is within the range of the genome sizes of *D. anilini* and NaphS2. An exception is SAG14, which by this procedure has a predicted genome size of only 1.7 Mbp. Large variation in genome size exists among known deltaproteobacterial sulfate reducers and for example the sulfate reducers *Desulfonauticus submarinus* DSM15269 and *Desulfomicrobium thermophilum* DSM16697 have genome sizes of 2.1 and 2.4 Mbp, respectively (IMG, genome IDs 2619619038 and 2571042920). Possibly SAG14 represents a distinct lineage with a small genome size, similar to other types of bacteria found in subsurface environment (Kantor et al., 2013). However, given the genome size range of its closest relatives it is more likely that the genome size of SAG14 is underestimated. The level of contamination in the SAGs was estimated by CheckM (Parks et al., 2015) and was overall very low with a maximum of 2% in SAG11 (**Table 1**). In agreement, we did not find conspicuously high numbers of putative horizontally transferred genes in the SAGs as compared to the genomes of *D. anilini* and strain NaphS2 using the IMG statistic for putative horizontally transferred genes (results not shown).

**Table 1:** Genome assembly statistics for SAGs and genomes of cultured *Desulfatiglans* members used for comparison.

		Assembly			Completeness			Contamination	
Name	Bigelow^#^ SAG name	IMG taxon ID	Assembly size (bp)	Number of genes	tRNAs (%)	quickCOAT^∗^ (%)	CheckM^§^ (%)	CheckM (%)	Lineage used for CheckM analysis
*D. anilini*	NA	2526164742	4,667,344	4,040	100	100	NA	NA	NA
NaphS2	NA	648276759	6,554,722	7,224	100	100	NA	NA	NA
SAG2	SCGC AB_539-E05	2703719328	1,037,801	1,094	15	18	20	1	Deltaproteobacteria
SAG3	SCGC AB_539-G04	2703719334	2,500,643	2,662	55	51	55	1.6	Deltaproteobacteria
SAG5	SCGC AB_540-A23	2703719263	826,310	848	32	21	19	0	Deltaproteobacteria
SAG8	SCGC AB_540-K05	2703719302	1,379,826	1,487	26	38	31	0.3	Deltaproteobacteria
SAG11	SCGC AD-745-C14	2710724210	1,132,095	1,212	19	19	20	2.1	Bacteria
SAG13	SCGC AD-745-L18	2710724212	1,551,347	1,548	15	27	25	1.7	Bacteria
SAG14	SCGC AD-745-M13	2710724213	855,461	940	36	45	50	0.6	Deltaproteobacteria

^#^*Single Cell Genomics Center, Bigelow Laboratory for Ocean Sciences, Maine (https://scgc.bigelow.org). ^∗^quickCOAT (Marshall et al., 2017b). The completeness estimate was based on the presence of conserved single copy genes as compared to the genomes of *Desulfobacterium anilini* and Deltaproteobacterium strain NaphS2. ^§^ CheckM (Parks et al., 2015)*.

### Phylogeny of SAGs and Abundance of *Desulfatiglans*-Related OTUs in Aarhus Bay Sediment

**FIGURE 1:**
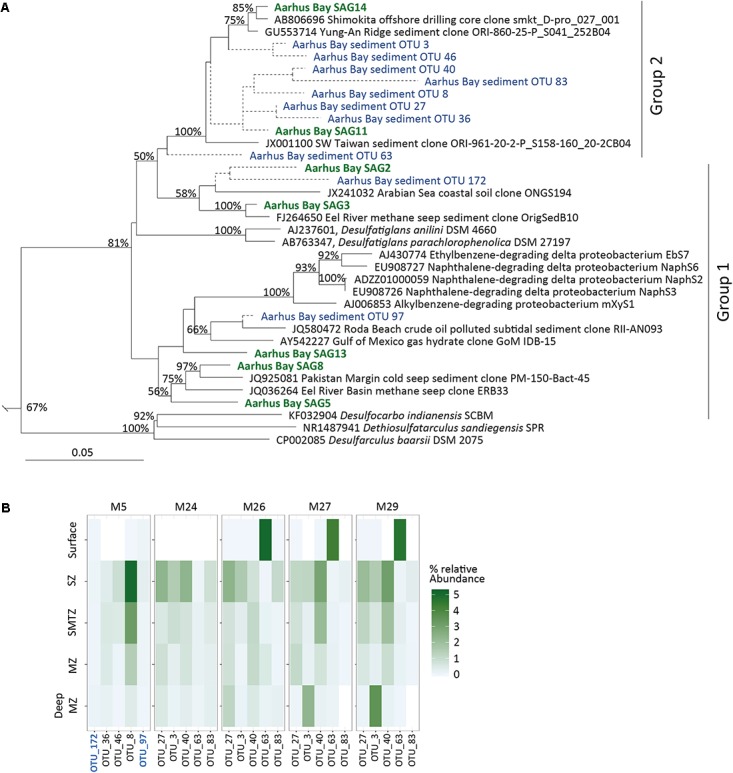
**(A)** Phylogenetic affiliation of 16S rRNA genes from SAGs and abundant *Desulfatiglans* OTUs from Aarhus Bay sediments. The tree was inferred by maximum likelihood analysis. SAGs are indicated in green and OTU sequences in blue. Aarhus Bay SAG2 and SAG11 as well as short OTU sequences were added to the tree without changing its overall topology (see main text for details) and are indicated by dashed lines. Numbers at nodes show bootstrap values (100 replications) above 50%. The scale bar shows 5% estimated sequence divergence. The tree is truncated to only show members of the family *Desulfarculaceae*, a full tree is available in the **Supplementary Figure S1**. **(B)** Heatmap of relative abundance of *Desulfatiglans*-related 16S rRNA gene sequence OTUs in amplicon libraries from different vertical geochemical sediment zones in Aarhus Bay sediment. SZ, sulfate zone; SMTZ, sulfate methane transition zone; MZ, methane zone. White fields in the heatmap indicate absence except for the surface of M24 for which data was not available. Group 1 OTUs are indicated in blue and bold font.

The 16S rRNA gene sequences of the single cells showed a strong phylogenetic affiliation with those of *D. anilini* and *Desulfatiglans parachlorophenolica* and the sulfate-reducing strains NaphS2, NaphS3, and NaphS6, mXyS1 and EbS7 (**Figure 1**). Together these sequences formed a clade with high (>80%) bootstrap support (**Figure 1A**). The SAGs clustered in three different lineages: SAG5, SAG8, and SAG13 clustered basal to strain NaphS2 and its related strains (mXyS1, EbS7, NaphS3, and NaphS6) while SAG2 and SAG3, and SAG11 and SAG14, respectively, formed individual lineages together with environmental taxa. The 16S rRNA gene sequences of the SAGs shared 89–94% identity to those of *D. anilini* and strain NaphS2 and shared 90–95% identity among each other (**Supplementary Table S3**). Based on established 16S rRNA gene sequence identity thresholds for taxonomic classification of uncultured taxa (Yarza et al., 2014) the members of this clade can thus be classified within the same family (threshold 86.5%), while the SAGs may represent several genera (threshold 94.5%). The SAGs, *D. anilini* and strain NaphS2 have pairwise ANI values ranging from 69 to 75% (**Supplementary Table S2**) which clearly delineate them all as different species (Richter and Rossello-Mora, 2009) but is within the range of ANI values characteristic for members of the same genus (Kim et al., 2014). Species that share less than 80% ANI and/or 30% of their gene content are considered too divergent to be compared with ANI and using average AAI provides a more robust resolution (Rodriguez-R and Konstantinidis, 2014). Based on AAI values all SAGs were within the genus distance of 60–80% AAI (Luo C. et al., 2014; Luo H. et al., 2014) from each other as well as *D. anilini* and NaphS2 (**Supplementary Table S2**). SAG11 and SAG14 were particularly distantly related to *D. anilini* and strain NaphS2 as they shared <90% pairwise 16S rRNA gene sequence identity (**Supplementary Tables S2**, **S3**). SAG11 and 14 were obtained from sulfate-depleted subsurface sediment, and we refer to them as “group 2,” to contrast them from the other SAGs which were obtained from sulfate-rich surface sediment and which we refer to as “group 1” (**Figure 1** and **Supplementary Table S1**).

The taxonomic classification of the genus *Desulfatiglans* is uncertain. Silva SSU releases 128 and 132 classifies strain NaphS2 and its relatives together with *D. anilini* and *D. parachlorophenolica* in the genus *Desulfatiglans*, and classifies this genus within the family *Desulfarculaceae* [order Desulfarculales]. In agreement, the 16S rRNA gene sequences of other members of this family (genera *Desulfarculus*, *Dethiosulfatarculus* and *Desulfocarbo*) form a sister lineage to those of the *Desulfatiglans*-related taxa (**Figure 1** and **Supplementary Figure S1**). According to NCBI taxonomy (accessed February 2018) the genus *Desulfatiglans* is classified within the family Desulfobacteraceae [order Desulfobacterales] while strains NaphS2, mXyS1, EbS7, NaphS3, and NaphS6 are delineated as unclassified Deltaproteobacteria and thus not assigned a taxonomic rank below the class-level. According to RDP taxonomy (RDP release 11, Cole et al., 2014) these strains are classified within the genus *Desulfatiglans* together with *D. anilini* and *D. parachlorophenolica*, but the genus is classified within the family Desulfobacteraceae. Notably, both the NCBI and RDP taxonomies recognize the family Desulfarculaceae and the order Desulfarculales but only classify *Desulfarculus*, *Dethiosulfatarculus*, and *Desulfocarbo* within these groups. Special caution should therefore be applied when evaluating the environmental distribution of *Desulfatiglans*-related taxa by high-throughput 16S rRNA gene amplicon sequencing because the results may strongly depend on the choice of reference database for the taxonomic classification.

We used the Silva SSU release 128 database for taxonomic classification of 16S rRNA gene amplicon sequence libraries generated from Aarhus Bay sediments and identified the five most frequent OTUs affiliated with the family *Desulfarculaceae* from each of two datasets (Marshall et al., 2017b; Starnawski et al., 2017). These datasets covered various sediments depths from five different sampling stations. Collectively these OTUs represented 0.1–7.1% of the total reads of the libraries. Eight of the 10 OTUs were most closely related to group 2 SAGs (**Figure 1**) sharing 93–96% sequence identity with SAG14 while the two remaining OTUs shared up to 95 and 96% sequence identity with group 1 SAGs, respectively (**Supplementary Table S3**). None of OTUs is closely related to the cultured members of the genus *Desulfatiglans* (**Figure 1** and **Supplementary Tables S2**, **S3**).

The sediment depth distribution of these OTUs suggest that *Desulfatiglans*-related populations are abundant members of the bacterial community throughout the sediment column with relative abundances of individual OTUs exceeding 1% of total bacteria in surface sediment and in sulfate-rich as well as sulfate-depleted subsurface sediment (**Figure 1** and **Supplementary Table S1**).

The *Desulfatiglans*-related populations in Aarhus Bay sediment are clearly phylogenetically different from their cultured relatives. These uncultivated populations seem to be adapted for subseafloor life as their relative abundance generally increases below the bioturbated surface (**Figure 1B**). The transition from the bioturbated surface sediment into the underlying undisturbed sediment has a strong influence on the size and composition of both the total microbial community and the SRM community in Aarhus Bay sediments (Chen et al., 2017; Jochum et al., 2017). This transition marks the beginning of the energy-deprived subsurface biosphere (Petro et al., 2017) where the *Desulfatiglans*-related populations appear to thrive relative to most other taxa.

### Group 1 SAGs Are Dissimilatory Sulfate Reducers That May Grow by Oxidation of Various Organic Carbon Substrates and Molecular Hydrogen

**FIGURE 2:**
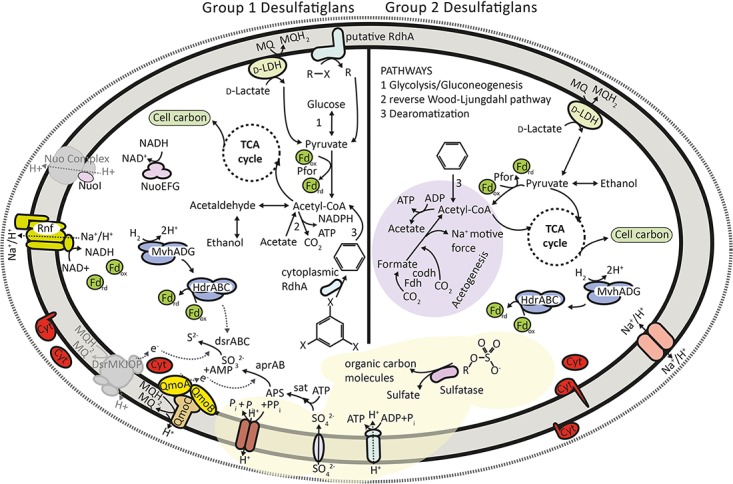
Overview of the metabolic potential encoded in group 1 and group 2 SAGs. Not all enzymes discussed in the text are shown for the sake of clarity. All chemical compounds shown are representative, the aromatic ring structure may represent any monoaromatic compound and halogenated organic compounds may represent any organohalide. Sulfate produced from sulfatases may be used in respiration in group 1 cells. Pfor, pyruvate:ferredoxin oxidoreductase; Fdh, NAD-dependent formate hydrogenase; Codh, carbon monoxide dehydrogenase; Cyt, multi-heme cytochrome; D-LDH, D-lactate dehydrogenase; MQ/MQH2, menaquinone pool; Fd, ferredoxin; Nuo, NADH:quinone oxidoreductase; Rnf, ferredoxin dependent transmembrane proton pump; Hdr, heterodisulfide reductase; Mvh, methyl viologen reducing hydrogenase; RdhA, reductive dehalogenase subunit A; PPi, pyrophosphate.

All group 1 SAGs contained genes encoding for proteins involved in the canonical dissimilatory sulfate reduction pathway, including sulfate transporters, ATP sulfurylase (*sat*), APS reductase (*aprAB*) and dissimilatory sulfite reductase (*dsrABC*) (**Figure 2**). The first step in dissimilatory sulfate reduction is the transport of the sulfate ion across the cell membrane, as it cannot enter the cell by passive diffusion. Typically sulfate permeases of the SulP family are annotated as the main sulfate transporter in SRM genomes (Pereira et al., 2011; Rabus et al., 2015). SulP homologs are encoded in *D. anilini* and strain NaphS2 genomes, but are not present in the SAGs. Genes encoding a SulT-type transporter, which may function as a sulfate transporter in some SRM (Rabus et al., 2015) was neither present in genomes of the two isolates nor in the SAGs. However, putative sulfate transporters (Marietou et al., 2018) belonging to the phosphate transporter family CysPit (PF01384) were detected in the SAGs (2, 3, 8), which are absent in *D. anilini* and *NaphS2* (**Supplementary Table S4**). SAG13 and SAG8 also possesses genes coding for homologs of sodium:sulfate transmembrane transporters (DASS family, PF00939), which are also found encoded in the genomes of *D. anilini* and NaphS2. Notably, a gene expression study suggests that DASS-family transporters function in high-affinity sulfate uptake in the SRM *Desulfobacterium autotrophicum* (Tarpgaard et al., 2017). SAG5 as well as *D. anilini* strain NaphS2 also carry a gene encoding a CysZ-family protein (COG2981) (**Supplementary Table S4**), which also could function in sulfate uptake in SRM (Marietou et al., 2018).

The reduction of sulfate to sulfite has a very low redox potential due to the stability of the sulfate ion (Thauer et al., 2007) and it requires activation with ATP to form adenosine phosphosulfate (APS) by the enzyme ATP sulfurylase (Sat) (Rabus et al., 2015). This endergonic reaction is driven by the hydrolysis of inorganic pyrophosphate (PPi) by a pyrophosphatase, which is a soluble protein in most SRM (Serrano et al., 2007). We could identify genes encoding Sat in three SAGs and found a proton translocating membrane bound pyrophosphatase in SAG3 (**Supplementary Table S4**) but indications for soluble pyrophosphatases were missing. The gene encoding membrane bound pyrophosphatase is also present in the genomes of strain NaphS2 and *D. anilini*, however, *D. anilini* additionally contains multiple genes annotated as cytoplasmic pyrophosphatases (Suzuki et al., 2014). Transmembrane ion-translocating pyrophosphatase is probably involved in energy conservation by establishing a proton gradient and power ATP production across the membrane (Serrano et al., 2007). This mechanism was previously also described for *Syntrophus gentianae*, a syntrophically benzoate-degrading fermenting bacterium (Schocke and Schink, 1998). As pyrophosphate is a by-product of various metabolic pathways its use for ATP production can provide an organism with essential energy reserves particularly during stress and low-energy conditions (Serrano et al., 2007), which is likely advantageous in energy-poor subsurface sediments.

The two key reductases, AprAB and DsrAB of the dissimilatory sulfate reduction pathway are soluble and thus not directly connected to membrane-associated electron transport. According to existing models, the membrane bound protein complexes, QmoABC and DsrMKJOP transfer electrons from the membrane quinone pool to AprAB and DsrAB, respectively (Pires et al., 2006; Ramos et al., 2012). The three subunits of the QmoABC membrane complex were encoded in SAG2, 5 and 8 adjacent to their respective *aprAB* genes (**Supplementary Table S4**), which is also the case for many known SRM (Ramos et al., 2012). Homologs of genes encoding the subunits of the DsrMKJOP complex were absent in all group 1 SAGs. As the *dsrMKJOP* genes are consistently located in a single operon in SRM and sulfide-oxidizing microorganisms (Venceslau et al., 2014) it is possible that DNA amplification was not successful in this genomic region and therefore we failed to detect these genes in the SAGs. Typically, DsrMKJOP is encoded next to DsrC (Venceslau et al., 2014). However, this is not the case in NaphS2 and likely not in the SAGs either as both SAG3 and SAG8 encode a DsrC/TusE-family protein (**Supplementary Table S4**). DsrC has a highly conserved C-terminal sequence and its conserved cysteine residues that distinguish DsrC from TusE (Venceslau et al., 2014) were present in the DsrC homolog encoded by both SAGs. DsrC has key role in the catalytic cycle of DsrAB (Santos et al., 2015) and is thus likely essential for SRM. The electron transfer between the membrane quinone pool and DsrAB could also be carried out by alternative complexes to DsrMKJOP, such as TmcABCD and HmcABCDEF (Pereira et al., 2011), but we could not detect the presence of genes encoding these complexes in the SAGs either. Collectively, the group 1 SAGs encode an otherwise complete set of proteins involved in dissimilatory sulfate reduction suggesting that members of this group conserve energy by this pathway.

SAG8 of group 1 encodes all subunits of the Rnf complex (**Supplementary Table S4**), which is an ion translocating electron transport complex found in several SRM (Biegel et al., 2011; Rabus et al., 2015) including also *D. anilini* and strain NaphS2. The Rnf complex is believed to mediate electron transfer between NADH and ferredoxin either conserving or dissipating energy depending on the direction of the electron transfer. However, the SAG8 Rnf gene operon also encodes a periplasmatic cytochrome c3 (**Supplementary Table S4**). This is typical for Rnf-carrying SRM and suggests that the Rnf complex also mediates electron transfer to/from the periplasm (Pereira et al., 2011). We found evidence for another potentially ion translocating electron transfer complex in SAG3, 5, and 8 with several subunits of complex 1 [Nuo, NADH:quinone oxidoreductase complex (Sazanov, 2014)], however, we could not detect the membrane spanning subunits and therefore the presence of this complex remains uncertain (**Figure 2**). A full Nuo complex is encoded in the genome strain NaphS2. In *D. anilini*, this complex seems to be partially present including only the cytoplasmic NuoEFG subunits that form the NADH dehydrogenase module of the complex.

Group 1 SAGs encode the genes for an enzymatic machinery capable of oxidizing various organic carbon substrates, suggesting a diverse heterotrophic metabolism (**Figure 2** and **Supplementary Table S5**). We could identify key genes of the Wood–Ljungdahl (reductive coenzyme-A) pathway in the group 1 SAGs (**Supplementary Table S5**). Among sulfate reducers this pathway is known to be involved in autotrophic CO_2_ fixation, acetogenesis and acetate oxidation operating either in the forward or reverse direction (Rabus et al., 2006, 2015). This pathway is also encoded in the genomes of *D. anilini* and strain NaphS2. These isolates are acetate oxidizers and have not been reported to grow autotrophically or by acetogenesis (Galushko et al., 1999; Schnell et al., 1989; Suzuki et al., 2014). Given that group 1 SAGs were derived from sulfate-rich surface sediments we find it more likely that the populations represented by these SAGs utilize the Wood–Ljungdahl pathway for acetate oxidation (**Figure 2**) similar to *D. anilini* and strain NaphS2. Furthermore, an alcohol dehydrogenase may enable group 1 cells to utilize alcohols, and pyruvate can be oxidized by pyruvate:ferredoxin oxidoreductase (Pfor), both processes leading to acetyl-CoA, which can either be used in the reverse Wood–Ljungdahl pathway, leading to energy conservation or be fed into an incomplete tricarboxylic acid (TCA) cycle to synthesize cell carbon (**Figure 2** and **Supplementary Table S5**). While the genomes of *D. anilini* and strain NaphS2 both encode a full set of enzymes involved in the canonical oxidative TCA cycle, we also detected a gene encoding 2-oxoglutarat lyase in SAG3 (**Supplementary Table S5**), indicating that the TCA cycle might be operating in both directions in group 1 *Desulfatiglans*. SAG13 encodes a flagellum system (**Supplementary Table S9**) unlike *D. anilini*, strain NaphS2 and the other SAGs. It also encodes chemotaxis proteins suggesting that this particular SAG may represent a population inhabiting the more dynamic surface sediment environment. In contrast, flagellar motility and chemotaxis is not considered important traits in subsurface sediments (Lever et al., 2015).

Cells of group 1 may oxidize lactate using a D-lactate dehydrogenase (D-LDH), found in SAG3 (**Figure 2** and **Supplementary Table S4**), which is a respiratory enzyme involved in electron transfer, located on the cytoplasmic side of the inner membrane (Dym et al., 2000). This type of lactate dehydrogenase is also present in *D. anilini*, however, it is not found in NaphS2. SAG3 and SAG8 carried genes encoding cytoplasmic iron-only hydrogenase (**Supplementary Table S4**), which are also present in *D. anilini* and NaphS2, however, no genes coding for periplasmic hydrogenases were found in any of the SAGs. *D. anilini* and NaphS2 both contain periplasmic hydrogenases. Even though the absence of periplasmatic hydrogenases in SRM is unusual, some SRM are known to function without those enzymes, indicating that hydrogen metabolism is not essential for sulfate reduction (Pereira et al., 2011; Rabus et al., 2015). Group 1 SAGs, however, encode all three subunits of a cytoplasmic [NiFe]-hydrogenase of the Mvh-type along with the cytoplasmic heterodisulfide reductase HdrABC (**Supplementary Table S4**). This complex is widespread in SRM and can couple the oxidation of H_2_ to the reduction of ferredoxin and DsrC (Kaster et al., 2011; Rabus et al., 2015) thus possibly facilitating hydrogenotrophic growth (**Figure 2**). As this complex is present in many anaerobes it might also have a more general function in anaerobic metabolism (Ramos et al., 2015) not coupled to sulfate reduction in the SAGs. Group 1 SAGs contained several multi-heme cytochromes (**Supplementary Table S4**) and fall thus in the cytochrome-rich group of SRM (Rabus et al., 2015) but we could not link their presence to a specific process. High numbers of multi-heme cytochromes are believed to be a characteristic of metabolically versatile anaerobes that have to adapt to changing conditions (Thomas et al., 2008), a trait that certainly could be of advantage in marine sediments as redox conditions gradually change during burial into deeper layers of sediment.

### Group 2 SAGs Show Potential for Conserving Energy by Acetogenesis and/or Fermentation but Likely Not Sulfate Reduction

Surprisingly, as their closest cultured relatives all grow by sulfate reduction, the two group 2 SAGs (SAG11 and 14) did not contain any genes involved in the dissimilatory sulfate reduction pathway, except that SAG14 encodes a CytPit-family protein putatively involved in sulfate uptake (**Figure 2** and **Supplementary Table S5**). Instead, we found evidence for an acetogenic and/or fermentative strategy for energy conservation in group 2 SAGs. Acetogens use the reductive acetyl-CoA (Wood–Ljungdahl) pathway for both energy conservation and assimilation of carbon (Bräsen et al., 2008; Ragsdale and Pierce, 2008). Similar to group 1 SAGs, group 2 SAG14 encodes several key enzymes of the Wood–Ljungdahl pathway (**Supplementary Table S5**). We further found a gene encoding a phosphate acetyltransferase, which previously was identified as one of two key enzymes for acetogenesis in Bathyarchaeota (He et al., 2016). In the lack of the capacity for dissimilatory sulfate reduction we therefore hypothesize that group 2 SAGs represent populations that conserve energy by acetogenesis in agreement with their presence in deep sulfate-depleted sediment. As discussed below, acetogenesis in group 2 cells is possible using reducing equivalents from the oxidation of fatty acids or alcohols or from degradation of aromatic compounds to reduce CO_2_.

Oxidation of lactate by lactate dehydrogenase, which produces pyruvate that in turn can be oxidized by Pfor, could provide reducing equivalents for the reductive acetyl-CoA pathway (**Figure 2**). Pfor is present in both group 2 SAGs (**Supplementary Table S5**). In acetogens pyruvate is typically produced from sugars via glycolysis (Ragsdale and Pierce, 2008), however, we did not detect genes encoding enzymes commonly associated with glycolysis in the group 2 SAGs. We cannot conclude on the absence of glycolysis with certainty due to partial genomes available for group 2 but if glycolysis is missing in those cells, pyruvate could, apart from oxidation of lactate, also be produced by oxidation of alcohols by alcohol dehydrogenase, which is present in SAG11 (**Supplementary Table S5**).

Acetogens are often metabolically flexible and can couple acetogenesis to other pathways such as pyruvate oxidation to acetyl-CoA by Pfor (Ragsdale and Pierce, 2008). Pfor also catalyzes the first step in the reductive TCA cycle, which is used by anaerobes to incorporate the acetyl group of acetyl-CoA into cell carbon and to generate metabolic intermediates. The reductive TCA cycle is distinct from its oxidative direction by the presence of three enzymes, fumarate reductase, 2-oxoglutarat lyase and ATP citrate lyase (Evans et al., 1966; Hugler et al., 2007). We found all enzymes indicative of reductive TCA cycle in SAG11 (**Supplementary Table S5**) suggesting that the TCA cycle enzymes are used for biosynthesis in group 2 SAGs. In many anaerobes an incomplete TCA cycle is used in both oxidative and reductive direction (Fuchs and Stupperich, 1978; Wood et al., 2004), which is an option for group 2 SAGs as SAG14 encodes citrate synthase (**Supplementary Table S5**) working in the oxidative direction of the TCA cycle. *D. anilini* and NaphS2 only possess the genes encoding an oxidative TCA cycle. The putative possibility for group 2 SAGs to use the cycle in both directions further points to their metabolic versatility as compared to cultured *Desulfatiglans* isolates.

Acetogens do not require a special set of electron transport related genes, suggesting that they can use electron transfer pathways from other metabolic cycles (Ragsdale and Pierce, 2008). We found cytoplasmic and periplasmic multi-heme cytochromes in both group 2 SAGs that potentially are involved in electron transport for acetogenesis (**Supplementary Table S4**). Generally, cytochrome c559, c554 and a menaquinone pool are believed to be part of the electron transfer chain in acetogens (Das et al., 1989) and both group 2 SAGs contained multiple c554 type cytochromes (**Supplementary Table S4**). To establish the membrane potential needed for chemiosmotic ATP synthesis group 2 SAG14 contains a sodium/proton ion translocating pyrophosphatase similar to group 1 SAGs (**Supplementary Table S4** and **Figure 2**). Furthermore, SAG14 encodes a sodium/proton ion transmembrane antiporter, which may provide channels for the proton pumping machinery (Moparthi et al., 2014). This gene is also present in NaphS2, however, not in *D. anilini*. The antiporter may be of importance in sodium-based ATP synthesis, which was suggested as a more efficient strategy for energy conservation (Lolkema et al., 1994) in deep subsurface sediments as compared to proton-driven ATP synthesis (Jørgensen and Marshall, 2016).

The identification of *Desulfatiglans*-related bacteria as acetogens adds evidence to the hypothesized role of acetogenesis as an important metabolic strategy in the subseafloor (Lever, 2012). Further, our results indicate that environmental abundance of sulfate reducers based on 16S rRNA taxonomy might have been overestimated in earlier studies. Environmental 16S rRNA sequence diversity affiliated to the genus *Desulfatiglans* were so far considered to represent SRM (Robador et al., 2016; Wasmund et al., 2017). However, our results challenge this conception with the inferred inability of group 2 SAG to grow by sulfate reduction. Other types of SRM are indeed also known to have close relatives that do not grow by sulfate reduction (Plugge et al., 2011).

We acknowledge that the incomplete genomes might complicate predictions of absence of metabolic pathways, however, in contrast to group 2 SAGs the group 1 SAGs of comparable completeness (**Table 1**) all contained at least one if not more genes involved in dissimilatory sulfate reduction.

The group 2 SAGs represent populations that are abundant in deep sulfate-depleted parts of Aarhus bay sediments. Interestingly, a recent study demonstrated that acetate is a key intermediate in organic matter degradation in such sulfate depleted marine sediments that fuels methanogenesis (Beulig et al., 2018). Radiotracer evidence suggested that acetate was not used directly by methanogens, but was oxidized by unknown syntrophic partners that pass reducing equivalents from acetate on to the methanogens. It is tempting to speculate the *Desulfatiglans* populations could represent such partner, in which case they would utilize the Wood–Ljungdahl pathway for acetate oxidation and bypassing the need for sulfate as electron acceptor for the process. Both group 1 and group 2 SAGs possess cytoplasmic hydrogenases that possibly could channel electrons into molecular hydrogen, which could subsequently fuel methanogenesis. Other interspecies electron transfer mechanisms involve direct transfers via outer membrane cytochromes as the case in sulfate-dependent anaerobic methane-oxidizing consortia (Wegener et al., 2015). Genes encoding such conserved outer membrane cytochromes (Skennerton et al., 2017) are, however, absent in the SAGs, suggesting also that the populations represented by the SAGs are not involved in anaerobic methane oxidation. Furthermore, genes previously identified important for syntrophic growth of SRM are also not found in the SAGs (Plugge et al., 2011).

### Reductive Dehalogenase Homologous Genes in *Desulfatiglans* and Relatives

**FIGURE 3:**
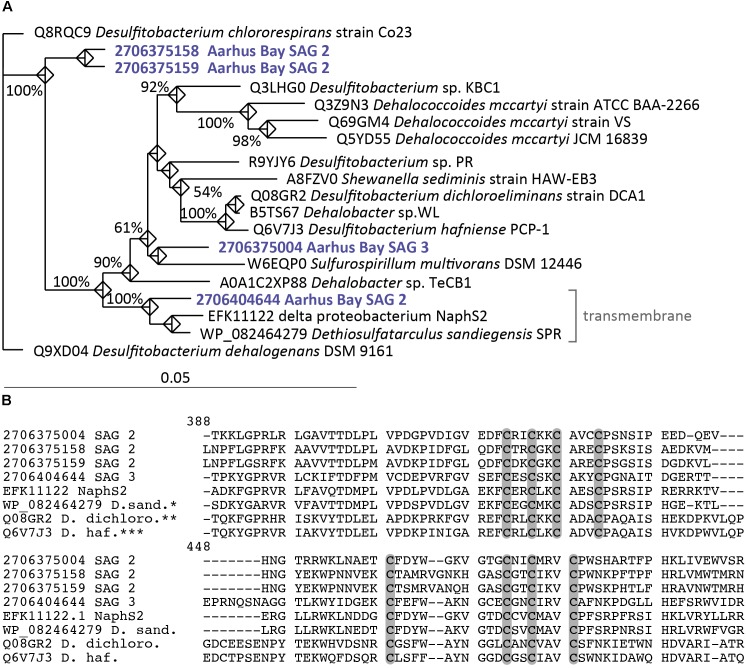
**(A)** Phylogenetic position of RdhA proteins from SAGs and known organohalide respiring microorganisms. Strain NaphS2 and *D. sandiegensis* have not been tested for organohalide respiration but have been added to the tree as closely related cultured representatives of the SAGs. Accession numbers and Gene IDs (for SAGs) are shown. The tree was inferred by maximum likelihood analysis. Numbers at nodes indicate bootstrap values (100 replications) above 50%. **(B)** RdhA amino acid alignment starting at position 388 (RdhA of *Desulfitobacterium dichloroeliminans* strain DCA1, Q08GR2) of conserved cysteine residues required for Fe–S cluster binding motifs typically found in RdhA. Cysteine residues are marked with gray bars. ^∗^D.sand., *Dethiosulfatarculus sandiegensis* SPR; ^∗∗^D.dichloro., *Desulfitobacterium dichloroeliminans* strain DCA1; ^∗∗∗^D.haf., *Desulfitobacterium hafniense* PCP-1.

Two of the group 1 SAGs contained homologs of *rdhA* genes, which encodes the main subunit of the reductive dehalogenase enzyme that catalyzes reductive dehalogenation of halogenated organic compounds, organohalogens (Schumacher et al., 1997) (**Supplementary Table S6** and **Figure 3**). Using reductive dehalogenases organohalide-respiring bacteria can use organohalogens as terminal electron acceptors, coupling reductive dehalogenation to respiratory energy conservation (Holliger et al., 1998; Atashgahi et al., 2016; Fincker and Spormann, 2017). Reductive dehalogenase enzymes are most extensively studied in *Dehalococcoides*, *Dehalobacter*, *Desulfitobacterium* strains and *Sulfurospirillum multivorans* (Jugder et al., 2016). RdhA of *Sulfurospirillum multivorans* is an Fe–S cluster-protein attached to the periplasmic side of the cytoplasmic membrane in a dimeric form with a putative membrane anchor protein (RdhB) (Bommer et al., 2014; Jugder et al., 2015; Fincker and Spormann, 2017). Organohalide-respiration involves electron transport via the menaquinone pool, however, the electron delivery to reductive dehalogenase enzymes has to date not been fully elucidated and the component connecting the menaquinone pool to reductive dehalogenase has not been identified (Jugder et al., 2016; Fincker and Spormann, 2017). SAG2 carried three different *rdhA* gene copies, two of which encode cytoplasmic proteins. Interestingly, the third *rdhA* gene of SAG2 encodes a transmembrane helix, which is also present in the closely related RdhA homologs encoded in strain NaphS2 and *D. sandiegensis* (**Supplementary Table S6** and **Figure 3A**) a cultured isolate within the family *Desulfarculaceae* (Davidova et al., 2015). Such *rdhA* genes encoding a transmembrane helix have to our knowledge not been described previously. Multiple non-identical copies of *rdhA* genes in one organism are known from *Dehalococcoides* strains and each paralog is likely specific for an individual type of organohalogen (Holscher et al., 2004; McMurdie et al., 2009). SAG3 contained one copy of the *rdhA* gene encoding a protein with a predicted cytoplasmic localization (**Supplementary Table S6** and **Figure 3A**). The predicted cytoplasmic and membrane-associated proteins in the SAGs as well as strain NaphS2 contained conserved cysteine residues required for Fe–S cluster binding (**Figure 3B**) typical for known RdhA enzymes, which suggests that the enzymes were functional (Wasmund et al., 2016). No genes encoding membrane-anchoring RdhB subunits were found in the SAGs. Similar *rdhA* homologs without signal peptides and missing associated RdhB subunits are encoded in the genomes of *Dehalococcoidia* (Fullerton and Moyer, 2016; Wasmund et al., 2016), which are another abundant group of microorganisms in Aarhus Bay subsurface sediments (Starnawski et al., 2017). The two cytoplasmic *rdhA* genes of SAG2 are located adjacent to each other and cluster phylogenetically separately from *rdhA* genes from known organohalide-respiring microorganisms, while the *rdhA* gene of SAG3 cluster with *rdhA* genes with a respiratory function (**Figure 3A**). The cytoplasmic *rdhA* genes found in SAG2 and SAG3 may not be part of a respiratory chain, as was previously suggested for the cytoplasmic RdhA homologs in Dehalococcoidia (Wasmund et al., 2016). Alternative functions suggested for non-respiratory dehalogenases could be reoxidation of respiratory cofactors, detoxification of substrates or removal of halogens from organics to enable downstream catabolism (Wasmund et al., 2016) as described for aerobic strains *Comamonas sp* strain 7D-2 and *Nitratireductor pacificus* pht-3B (Chen et al., 2013; Payne et al., 2015). However, the membrane associated protein in SAG2, NaphS2 and *D. sandiegensis* could represent a novel type of respiratory reductive dehalogenase, which instead of having a separate membrane anchor protein, is directly connected to the membrane with transmembrane helices. Neither NaphS2 nor *D. sandiegensis* is reported to grow by organohalide respiration (DiDonato et al., 2010; Davidova et al., 2015) complicating a functional prediction. The finding of *rdhA* genes in abundant subseafloor *Dehalococcoidia* (Fullerton and Moyer, 2016; Wasmund et al., 2016) as well as their high frequency in subseafloor metagenomes (Kawai et al., 2014; Gaboyer et al., 2015; Marshall et al., 2017a) suggest that the capacity to degrade or respire organohalogens is of competitive advantage in marine subsurface environments.

### Marine *Desulfatiglans* Encode Different Pathways for Aromatic Compound Degradation

Aromatic compound degradation might provide both acetogenic and sulfate-reducing *Desulfatiglans* populations with a non-competitive substrate as this class of compounds is generally inaccessible to most anaerobic microorganisms (Ahn et al., 2009; DiDonato et al., 2010; Lever, 2012). The biggest catalytic challenge microorganisms have to overcome in aromatic compound degradation is the stabilizing resonance energy of the aromatic ring system (Fuchs et al., 2011). Especially carboxylation reactions play a crucial role in activating and destabilizing of aromatic compounds during anoxic degradation. The best-characterized carboxylase involved in the anaerobic activation of aromatic compounds is phenyl phosphate synthase and phenylphosphate carboxylase (Boll et al., 2014) which is involved in phenol degradation in *D. anilini* (Ahn et al., 2009). Indeed, we could identify homologs of these enzymes in SAG8 (**Supplementary Table S7**), which are also present in the genomes of *D. anilini* and NaphS2. Enzymes for other activation mechanisms such as hydroxylation or fumarate addition (Fuchs et al., 2011; Boll et al., 2014) or the genes described for activation of polycyclic aromatic compounds in NaphS2 (DiDonato et al., 2010) could not be identified in any of the SAGs. We could not detect any genes involved in polycyclic aromatic compound degradation using the NaphS2 proteins described to catalyze this process as queries for blastp searches against the SAGs. Therefore, the results presented here refer to degradation of monocyclic aromatic compounds. Activation of aromatic compounds eventually leads to the central intermediate benzoyl-CoA or its derivatives (Fuchs et al., 2011; Ladino-Orjuela et al., 2016). The pathways for degradation of benzoyl-CoA differ essentially between an ATP-dependent and an ATP-independent pathway, with only the latter considered energetically feasible under anaerobic conditions (Fuchs et al., 2011). Type I benzoyl-CoA reductase (BCR I, BcrABC) depends on ATP to catalyze dearomatization, while the non-homologous type II benzoyl-CoA reductase (BCR II, BamBDEFGHI) uses an ATP-independent mechanism probably involving an electron bifurcation process to catalyze dearomatization (Fuchs et al., 2011; Boll et al., 2014). In agreement, *D. anilini* possesses a BCR II enzyme for dearomatization, but the strain NaphS2 genome encodes both BCR I and II and interestingly the BCR I is involved in naphthalene degradation (DiDonato et al., 2010). Similarly, the hyperthermophilic archaeon *Ferroglobus placidus* reportedly relies on a BCR I enzyme for anaerobic benzene degradation (Holmes et al., 2011). This indicates that the ATP-dependent pathway for dearomatization can be relevant also in strict anaerobes.

In SAG8, we found BamBCDE encoded in one operon, while interestingly SAG2 and SAG14 encoded the type I BcrA but not the type II (**Supplementary Table S7**). After the initial step of benzoyl-CoA breakdown by BCR enzymes, the pathway further proceeds through sequential removal of the double bonds and ring cleavage carried out by dienoyl-CoA hydratase (Dch), hydroxylacyl-CoA dehydrogenase (Had) and oxoacyl-CoA hydrolase (Oah) for type I BCR and enzymes encoded by *bamR*, *bamQ*, and *bamA* genes for type II BCR (Fuchs et al., 2011). For this part of the pathway we could detect Dch, Oah, and Had in SAG14 (**Supplementary Table S7**), suggesting that group 2 single cells carry a full set of genes for ATP-dependent degradation of aromatic compounds. We were unable to detect *bamR*, *bamQ*, and *bamA* homologs in the group 1 SAGs. Both group 1 (SAG3, 8) and group 2 (SAG11) SAGs encode non-decarboxylating glutaryl-CoA dehydrogenase (GDH) (**Supplementary Table S7**), which performs a key step in the degradation of benzoyl-CoA after dearomatization that can be coupled to energy conservation in obligate anaerobes (Wischgoll et al., 2009). The use of non-decarboxylating GDH is of special importance in sulfate-reducing and fermenting bacteria due to the energy constraints those groups face as compared to, e.g., denitrifying bacteria, which typically use a decarboxylating GDH that cannot conserve energy (Wischgoll et al., 2009). This non-decarboxylating enzyme is also present in NaphS2 and *D. anilini*. SAG8 and SAG14 along with the genomes of *D. anilini* and strain NaphS2 furthermore encode a homolog of cyclohexane-1-carbonyl-CoA dehydrogenase (SAG8 and 14 locus tags: Ga0167621_100321, Ga0151390_100391), which is a key enzyme for benzoate degradation in *Syntrophus aciditrophicus* (Kung et al., 2013). SAG3 encodes proteins homologous (∼40% AAI) to the products of the bbs gene cluster involved in toluene degradation in *Aromatoleum aromaticum* EbN1 (Kube et al., 2004) and naphthalene degradation in strain NaphS2 (DiDonato et al., 2010) (**Supplementary Table S7**). In summary this suggests that both group 1 and group 2 SAGs carry the genetic potential to degrade monoaromatic compounds. It is, however, puzzling how group 2 members may conserve energy by aromatic compound degradation using an ATP-consuming pathway in their energy-depleted subsurface environment. It is questionable whether this pathway is active under these conditions and we cannot exclude that group 2 members also carry BCR II type proteins that we did not detect due to the incompleteness of the SAGs. Alternatively, group 2 SAGs may encode BCR type II proteins that are more distantly related to known BCR type II proteins. We could identify genes putatively encoding BamBCDE proteins in SAG 14 (**Supplementary Table S7**), however, cannot conclude that these indeed represent *bona fide* BCR type II coding genes.

### Sulfatases May Provide Marine *Desulfatiglans* With Organic Carbon and Sulfite

Both group 1 and 2 SAGs encoded proteins with a predicted sulfatase activity (**Supplementary Table S8**). Additionally, genes encoding sulfatase-maturating enzymes, which are essential for post-translational modification and functionality of sulfatases (Benjdia et al., 2011) were identified in the group 1 SAGs (**Supplementary Table S8**). These genes were lacking in *D. anilini* and strain NaphS2 and may represent an adaptation to the sulfate- and organic-carbon-poor subsurface sediment environment. Sulfatases catalyze the removal of sulfate groups from various organic compounds, which allows the marine *Desulfatiglans* to access organosulfate compounds as an organic carbon source and a source of sulfate released from the hydrolysis of sulfate esters (Kertesz, 2000). The latter may represent an adaptation to sulfate limitation in the absence of preferred sulfur sources such as sulfate, sulfite, sulfide or cysteine for assimilatory or dissimilatory purposes (Kertesz, 2000). Organosulfur compounds can be particularly abundant in coastal marine environments (Glockner et al., 2003) and genes encoding sulfatases are frequently found in marine sediment metagenomes (Quaiser et al., 2011). Organic sulfur can be a significant source of sulfate especially in low sulfate sediment environments (King and Klug, 1980; Fakhraee et al., 2017) and can fuel up to 50% of sulfate reduction in marine sediments by *in situ* production of sulfate (Fakhraee et al., 2017). Abundant marine subsurface Dehalococcoidia, which are well represented in Aarhus Bay sediments (Wasmund et al., 2014; Starnawski et al., 2017) also encode sulfatases in their genome (Wasmund et al., 2014) suggesting that utilization of sulfonated organic compounds is a common feature among abundant subsurface microorganisms.

## Conclusion

Our results demonstrate that marine uncultured *Desulfatiglans*-related populations have a versatile metabolic potential. One group (group 1) of lower relative abundance in Aarhus Bay subsurface sediments has a sulfate-reducing lifestyle, while the second group (group 2), which represents dominant members of the bacterial community in subsurface sediments seems to be unable to perform dissimilatory sulfate-reduction. Instead its members may conserve energy by acetogenesis and/or fermentation. Members of both groups share the capability to access a wide range of carbon substrates including aromatic compounds, organosulfonates, and organohalides. Our results show that environmental *Desulfatiglans* cannot readily be considered sulfate-reducers, as was previously assumed, and that acetogenesis may be a key metabolic strategy for survival in energy- and sulfate- limited subsurface sediments.

## Availability of Data

Single-cell amplified genome assemblies were uploaded to the Integrated Microbial Genomes portal (https://img.jgi.doe.gov) with the following taxon IDs (2703719328, 2703719334, 2703719263, 2703719302, 2710724210, 2710724212, and 2710724213).

## Author Contributions

LJ, KK, and AS designed the research. LJ, LS, and IM performed the research. LJ and KK analyzed data and wrote the paper with input from all coauthors.

## Conflict of Interest Statement

The authors declare that the research was conducted in the absence of any commercial or financial relationships that could be construed as a potential conflict of interest.

## Footnotes

1 http://enve-omics.ce.gatech.edu/aai/index

2 https://www.ebi.ac.uk/Tools/msa/tcoffee/

## Supplementary Material

FIGURE S1 Phylogenetic affiliation of 16S rRNA genes from SAGs and abundant *Desulfatiglans* OTUs from Aarhus Bay. The tree was inferred by maximum likelihood analysis. Single cells are indicated in green and OTU sequences in blue. Aarhus Bay SAG2 and SAG11 as well as short OTU sequences were added to the tree without changing its overall topology (see main text for details) and are indicated by dashed lines. Numbers at nodes show bootstrap values (100 replications). The scale bar shows 10% estimated sequence divergence.

TABLE S1 Sediment sampling station data for single cell sorting and 16S rRNA amplicon sequencing and relative abundance of 16S rRNA gene sequence OTUs from PCR amplicon libraries generated from the sediments. ^∗^Latitude-Longitude. M5: 56.103333-10.457833. Mimosa: 56° 9′ 35.889 N, 10° 28′ 7.893 E. M24: 56.111883-10.41635. M26:56.11155-10.41715. M27A: 56.111317-10.41805. M29A: 56.11075- 10.419583. SZ, sulfate zone; SMTZ, sulfate methane transition zone; MZ, methane zone.

TABLE S2 Pairwise 16S rRNA % dissimilarity between cultured *Desulfatiglans* isolates and SAGs as well as pairwise average nucleotide identity (ANI) and average amino acid identity (AAI) values. ^∗^BBH, pairwise bidirectional best nSimScan hits (Markowitz et al., 2014).

TABLE S3 OTU 16S rRNA amplicon sequence and short SAG 16S rRNA sequence identity shared with cultured *Desulfatiglans* and full length 16S rRNA sequences of SAGs.

TABLE S4 Locus tags for genes involved in dissimilatory sulfate reduction, sulfate transport, and the electron transport chain in groups 1 and 2 SAGs.

TABLE S5 Locus tags for genes involved in carbon metabolism in groups 1 and 2 SAGs.

TABLE S6 Locus tags for *rdhA* genes in groups 1 and 2 SAGs.

TABLE S7 Locus tags for genes encoding enzymes involved in aromatic compound degradation in groups 1 and 2 SAGs. Locus tags in italics indicates a putative gene.

TABLE S8 Locus tags of genes encoding sulfatases or sulfatase-maturating factors in group 1 and 2 SAGs.

TABLE S9 Locus tags of genes encoding flagellar proteins and proteins involved in chemotaxis in groups 1 and 2 SAGs.
