# Supplementary figures and images for: Single-Cell Genomics Reveals a Diverse Metabolic Potential of Uncultivated Desulfatiglans-Related Deltaproteobacteria Widely Distributed in Marine Sediment
Source: Front Microbiol. 2018 Sep 3;9:2038. doi: 10.3389/fmicb.2018.02038 (PMC6129605; doi:10.3389/fmicb.2018.02038)

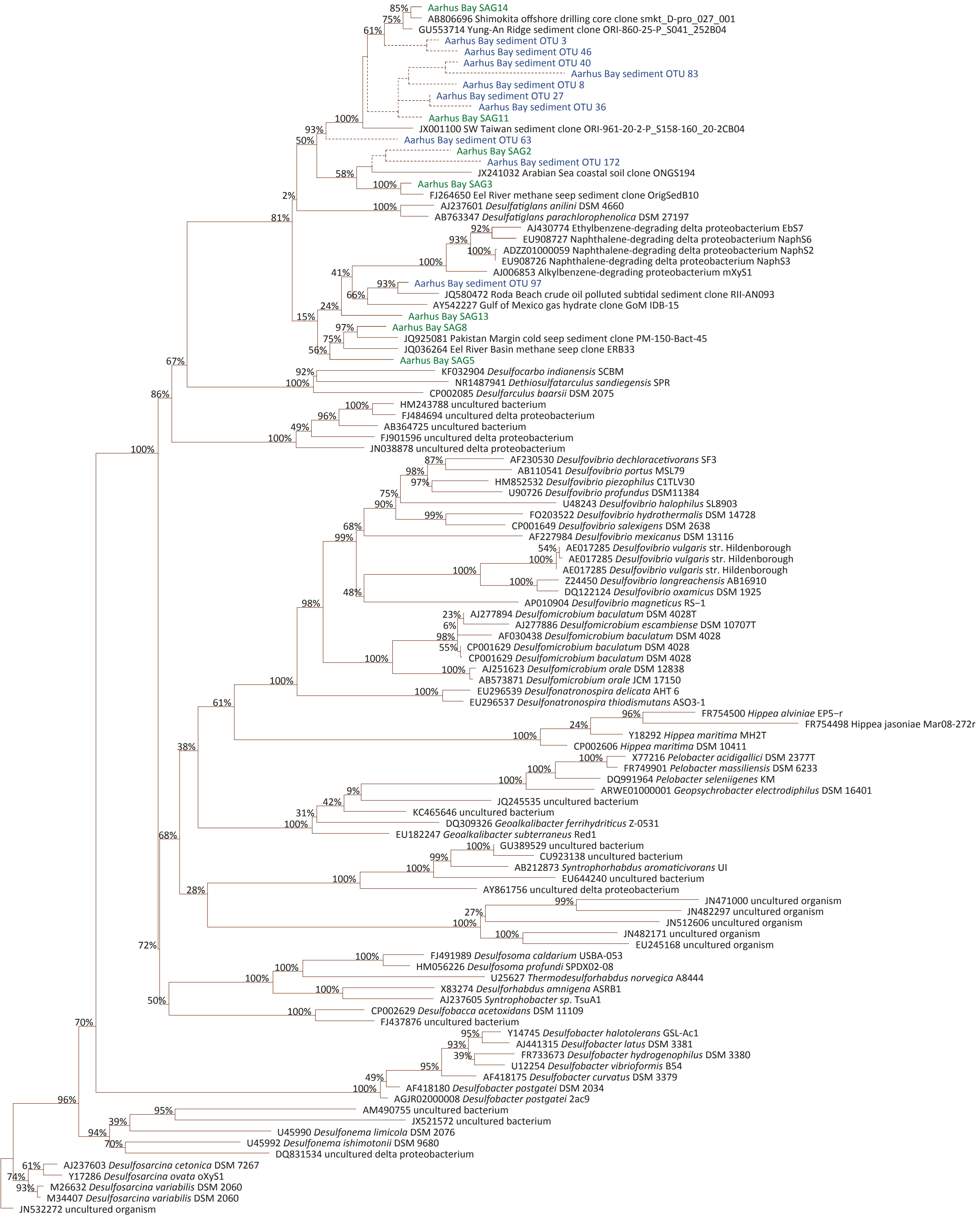

Supplement: FIGURE S1 — Phylogenetic affiliation of 16S rRNA genes from SAGs and abundant Desulfatiglans OTUs from Aarhus Bay. The tree was inferred by maximum likelihood analysis. Single cells are indicated in green and OTU sequences in blue. Aarhus Bay SAG2 and SAG11 as well as short OTU sequences were added to the tree without changing its overall topology (see main text for details) and are indicated by dashed lines. Numbers at nodes show bootstrap values (100 replications). The scale bar shows 10% estimated sequence divergence. [file Data_Sheet_1.PDF]
